# DegronMD: Leveraging Evolutionary and Structural Features for Deciphering Protein-Targeted Degradation, Mutations, and Drug Response to Degrons

**DOI:** 10.1093/molbev/msad253

**Published:** 2023-11-22

**Authors:** Haodong Xu, Ruifeng Hu, Zhongming Zhao

**Affiliations:** Department of Orthopaedics, The Second Xiangya Hospital, Central South University, Changsha, Hunan 410011, China; Center for Precision Health, School of Biomedical Informatics, The University of Texas Health Science Center at Houston, Houston, TX 77030, USA; Center for Precision Health, School of Biomedical Informatics, The University of Texas Health Science Center at Houston, Houston, TX 77030, USA; Center for Advanced Parkinson Research, Brigham and Women's Hospital, Harvard Medical School, Boston, MA 02115, USA; Genomics and Bioinformatics Hub, Department of Neurology, Brigham and Women's Hospital, Harvard Medical School, Boston, MA 02115, USA; Center for Precision Health, School of Biomedical Informatics, The University of Texas Health Science Center at Houston, Houston, TX 77030, USA; MD Anderson Cancer Center UTHealth Graduate School of Biomedical Sciences, Houston, TX 77030, USA; Human Genetics Center, School of Public Health, The University of Texas Health Science Center at Houston, Houston, TX 77030, USA

**Keywords:** protein-targeted degradation, ubiquitin–proteasome system, degron, evolutionary analysis, drug responses, machine learning, database

## Abstract

Protein-targeted degradation is an emerging and promising therapeutic approach. The specificity of degradation and the maintenance of cellular homeostasis are determined by the interactions between E3 ubiquitin ligase and degradation signals, known as degrons. The human genome encodes over 600 E3 ligases; however, only a small number of targeted degron instances have been identified so far. In this study, we introduced DegronMD, an open knowledgebase designed for the investigation of degrons, their associated dysfunctional events, and drug responses. We revealed that degrons are evolutionarily conserved and tend to occur near the sites of protein translational modifications, particularly in the regions of disordered structure and higher solvent accessibility. Through pattern recognition and machine learning techniques, we constructed the degrome landscape across the human proteome, yielding over 18,000 new degrons for targeted protein degradation. Furthermore, dysfunction of degrons disrupts the degradation process and leads to the abnormal accumulation of proteins; this process is associated with various types of human cancers. Based on the estimated phenotypic changes induced by somatic mutations, we systematically quantified and assessed the impact of mutations on degron function in pan-cancers; these results helped to build a global mutational map on human degrome, including 89,318 actionable mutations that may induce the dysfunction of degrons and disrupt protein degradation pathways. Multiomics integrative analysis unveiled over 400 drug resistance events associated with the mutations in functional degrons. DegronMD, accessible at https://bioinfo.uth.edu/degronmd, is a useful resource to explore the biological mechanisms, infer protein degradation, and assist with drug discovery and design on degrons.

## Introduction

Precise spatial and temporal regulation of protein degradation is indispensable for many cellular processes, including cell cycle progression, signaling, differentiation, and growth, whereas its dysregulation has been implicated in almost all hallmarks of cancer (Ravid and Hochstrasser 2008; Koren et al. 2018; Pohl and Dikic 2019). In recent years, rapid advancements in technologies for targeted protein degradation, such as proteolysis-targeting chimeras (PROTACs), have unveiled potential drug targets among previously considered nondruggable proteins, providing new approaches for drug discovery and design (Khan et al. 2019; Sun et al. 2019; Békés et al. 2022).

It is worth noting that more than 80% of intracellular protein degradation is predominantly governed by the ubiquitin–proteasome system (UPS; Ciechanover 2015; Xu et al. 2017, 2021). The UPS relies on E3 ubiquitin ligases and degrons, which are short linear motifs integrated within modular protein sequences and utilized by E3 ligases to target specific proteins (Pan et al. 2021). One crucial characteristic of degrons is their transferability; in most cases, transferring a degron from an unstable protein into a target protein accelerates the degradation of the latter, making it a promising approach for targeted protein degradation (Hou et al. 2022). Consequently, a systematic investigation of degrons and their specific relationship with the mediated E3 substrates is important for understanding their biological mechanisms, their implications in protein degradation, and discovery of potential therapeutic targets.

Dysfunction of degrons disrupts protein degradation pathways, leading to the abnormal accumulation of proteins, which can initiate and drive disease progression, especially in cancer (Mészáros et al. 2017; Martínez-Jiménez et al. 2020; Hu et al. 2021; Liu et al. 2021; Tokheim et al. 2021; Welcker et al. 2022). Cancer-related mutations linking to degron dysfunction have been identified in multiple oncoproteins, such as transcription factors MYC and NRF2 (Villeneuve et al. 2013), as well as various mitotic receptors like NOTCH1 and several receptor tyrosine kinases. Notably, degron dysfunction resulting from β-catenin mutations has been reported in numerous cancer types and is recognized as one of the most pivotal driver mutations in cancer (Mészáros et al. 2017). Mutations leading to degron dysfunction can alter the effectiveness of drug therapy, resulting in drug resistance. For instance, the drug lenalidomide demonstrates clinical efficacy against multiple myeloma and other B cell tumors (Jan et al. 2021). Lenalidomide induces the ubiquitination and degradation of 2 crucial transcription factors, IKZF1 and IKZF3, through the CRBN-CRL4 ubiquitin ligase. Mutations in the degron of IKZF3 disrupt its degradation signaling, leading to resistance to lenalidomide therapy (Krönke et al. 2014). While these studies have suggested the importance of degrons in drug treatment, they only represent a limited number of case studies. Given the availability of large-scale proteomics and genomics data sets, there is an urgent need for a comprehensive mutational landscape of the degrome to offer researchers a vital resource for investigating cellular homeostasis and potential drug responses associated with degrons.

**Fig. 1.**
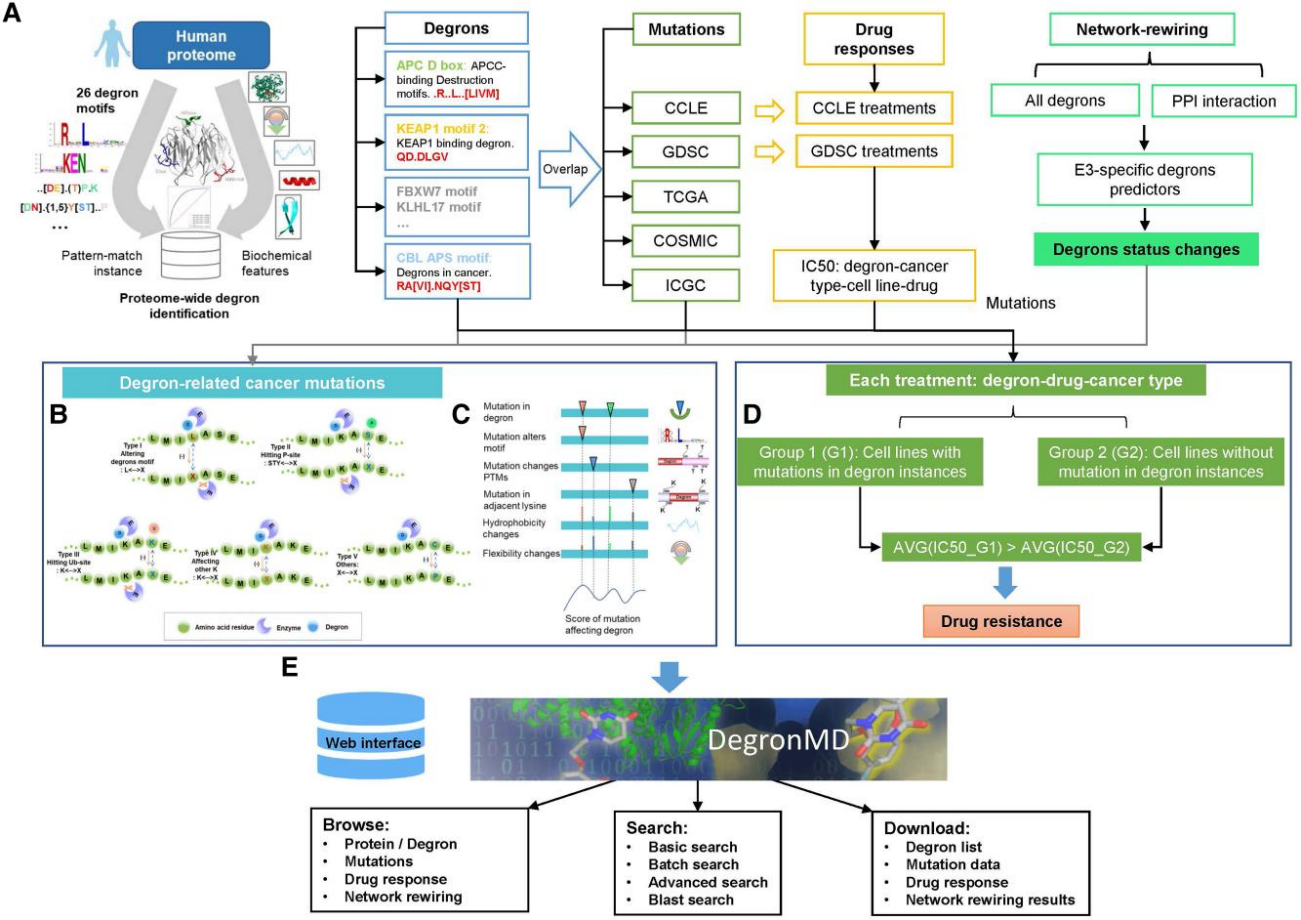
DegronMD workflow for data collection, processing, methodology, and database construction. a) Identification of new degrons in the human proteome through pattern recognition and machine learning technique. b) Five functional outcomes for degron-related mutations. c) DegMF method for quantifying the impact of missense mutations on degron function based on the observed phenotypic changes. d) The strategy for detecting drug resistance potentially caused by degron mutations. e) Construction of DegronMD database.

The human genome encodes over 600 E3 ubiquitin ligases, yet only a limited number of specific degron instances have been identified by well-defined enzymes (Morreale and Walden 2016). The limited number of known degrons presents a formidable challenge in systematically analyzing and detecting dysfunctional events resulting from degron mutations. To address these challenges, we developed DegronMD, an open knowledgebase designed for annotating new degrons across the human proteome for degradation, and it also provides the mutational landscape on degrome and associated drug response, including drug resistance (Fig. 1). In brief, our curated data revealed that degrons exhibited evolutionary conservation and were predominantly found in proximity to posttranslational modification (PTM) sites, particularly within disordered protein regions with higher solvent accessibility. Leveraging the inherent properties of degrons, we developed a machine learning approach, named DegML (*Deg*ron prediction based on *M*achine *L*earning), to predict more than 18,000 new degrons within the human proteome, incorporating consensus motifs and multiple intrinsic protein structural and evolutionary features. By quantitatively assessing the impact of mutations on key degron characteristics, we unveiled a comprehensive mutational landscape within the degrome, identifying 89,318 actionable mutations that directly disrupt UPS homeostasis by altering degron motifs and interfering with critical PTM signals. Furthermore, multiomics analysis revealed a landscape of drug resistance–associated mutations in functional degrome. All of the analyzed data are readily accessible through the DegronMD database (https://bioinfo.uth.edu/degronmd), which offers a wide array of functions, including advanced search, browsing, and visualization tools to explore protein degradation signals, mutations, and drug responses. We anticipate that DegronMD will serve as a valuable resource for the clinical and translational community, facilitating the investigation of protein homeostasis regulation, cancer research, and drug development.

## Materials and Methods

### Data Collection and Processing

We first collected and processed human degron motifs, i.e. consensus patterns for a specific E3 ubiquitin ligase, from the ELM database (Kumar et al. 2020) and another study (Mészáros et al. 2017). Degron motifs that were not specifically relevant to ubiquitination were removed. In our analysis, only internal motifs were used, resulting in 23 distinct motifs and a total of 133 degron instances (supplementary table S1, Supplementary Material online). As an example, the consensus motifs of APC/D box can be formatted as RxxLxx[LIVM], where x represents any of the 20 types of amino acids. Somatic mutations from various cancer genomic databases, including TCGA (Liu et al. 2018), COSMIC (Tate et al. 2019), ICGC (Zhang et al. 2019), CCLE (Ghandi et al. 2019), and GDSC (Yang et al. 2012), were acquired for pan-cancer analysis. We exclusively considered nonsynonymous mutations (i.e. changes of amino acids). Subsequently, all collected nonsynonymous mutations were mapped to the UniProt protein library. Following the elimination of synonymous mutations and mismatched records, we assembled a curated data set of over 7.2 million somatic mutation records. These mutations were then correlated with each degron and its flanking regions, extending 11 amino acids in both directions. They were classified into 5 distinct categories based on their functional effects on degrons: rewiring degron network, altering degron motif, blocking phosphorylation regulatory signal, blocking ubiquitination regulatory signal, and substituting flanking lysine.

For the multiomics analysis, we obtained experimentally verified PTM information from our constructed Eukaryotic Phosphorylation Sites Database (EPSD; Lin et al. 2021) and Protein Lysine Modifications Database (PLMD; Xu et al. 2017) resources, respectively. The EPSD database integrates 1,616,804 experimentally validated phosphorylation sites. The PLMD database contains 284,780 lysine modification events on 53,501 substrates, including 20 modification types. We also downloaded gene expression and DNA methylation profiles from the TCGA resource (Liu et al. 2018) for pan-cancer analysis to further explore the potential therapeutics and biomarkers of cancers. DNA methylation levels were determined by Illumina Infinium Human Methylation 450K platform, and gene expression profiles were measured via the Illumina HiSeq 2000 RNA Sequencing platform.

### Model Construction and Degron Prediction

To better characterize degron, we constructed a background data set containing a large number of randomly selected length-matched peptides on the same degron substrates. Based on curated degrons and random peptides, multiple evolutionary, structural, and physicochemical properties were investigated. Later, we trained 10 XGBoost classifiers using the bootstrapping strategy based on multiple features between degrons and random peptides. To determine the best parameters for each model, we tested dozens or hundreds of different parameter combinations for each model and selected the optimal parameters through multiple cross-validation (CV) evaluations. The average prediction scores of these 10 models determine the final probability of degron. To ensure that the model can make predictions when the protein features are not matched, we further extended the model using deep learning technology. Sequence of all degrons and their surroundings was intercepted (50-amino-acid length) and was transformed into 1,024-dimensional embedding by the pretrained protein language model of SeqVec (Heinzinger et al. 2019), which could accurately depict the physical properties of every single amino acid. These vectors are subsequently fed into 2 dense layers: the first of which has 128 hidden neurons that are activated by RELU and the second of which contains 64 such neurons. The final output layer has a single neuron, which provides the possibility of a degron. The final model, named DegML, was used to predict proteome-wide degrons.

### Drug Resistance Analysis in Degrome

Mutations may decrease drug sensitivity or lead to drug resistance through affecting degron. First, mutations can generate specific substrate proteins, such as oncogenes and kinases, that are more stable and lead to resistance to drugs or kinase inhibitors by altering key residues on the degron motifs, disrupting degradation signals. Second, mutations that occur on degrons can cause structural changes. For example, it changes the residue from a small side chain that can hold the drug spatially to a large side chain that hinders the binding of the drug and protein, leading to drug resistance. Finally, mutations on degrons may alter the structural environment such as solvent accessibility, disorder, and flexibility, resulting in small-molecule drugs that are less likely to bind to target proteins and, thus, trigger drug resistance. The CCLE project comprehensively characterized numerous human cancer cell lines using genetic and pharmacologic techniques, granting public access to the drug response data of 24 drugs across a collection of over 1,000 cell lines (Ghandi et al. 2019). Similarly, the GDSC project investigated drug response by administering 265 anticancer drugs to a population exceeding 1,000 cancer cell lines, each with their own somatic mutation profiles (Yang et al. 2012). In order to investigate the potential mutations associated with drug response in cancer, the raw drug response data concerning degron proteins, along with the corresponding cell line annotation files (CCLE cell lines, GDSC cell lines), were collected from both the CCLE and GDSC projects. Subsequent analysis focused solely on drug treatments targeting degron proteins.

### Database Construction

All the processed and analyzed data, along with the rich annotations, are stored and available in a new database, DegronMD (Fig. 1e). The dynamic web pages were implemented by utilizing several JavaScript libraries and Ajax strategies. The web interface of DegronMD was implemented using PHP and Bootstrap 4 (http://getbootstrap.com/). The visualization of interactive charts was created by using highcharts (https://www.highcharts.com/). We provided 5 manners for users to easily browse the data and 3 options to search the database. A tutorial page is provided at https://bioinfo.uth.edu/degronmd/tutorial.html.

## Results

### Proteome-Wide Analysis and Identification of Degrons

**Fig. 2.**
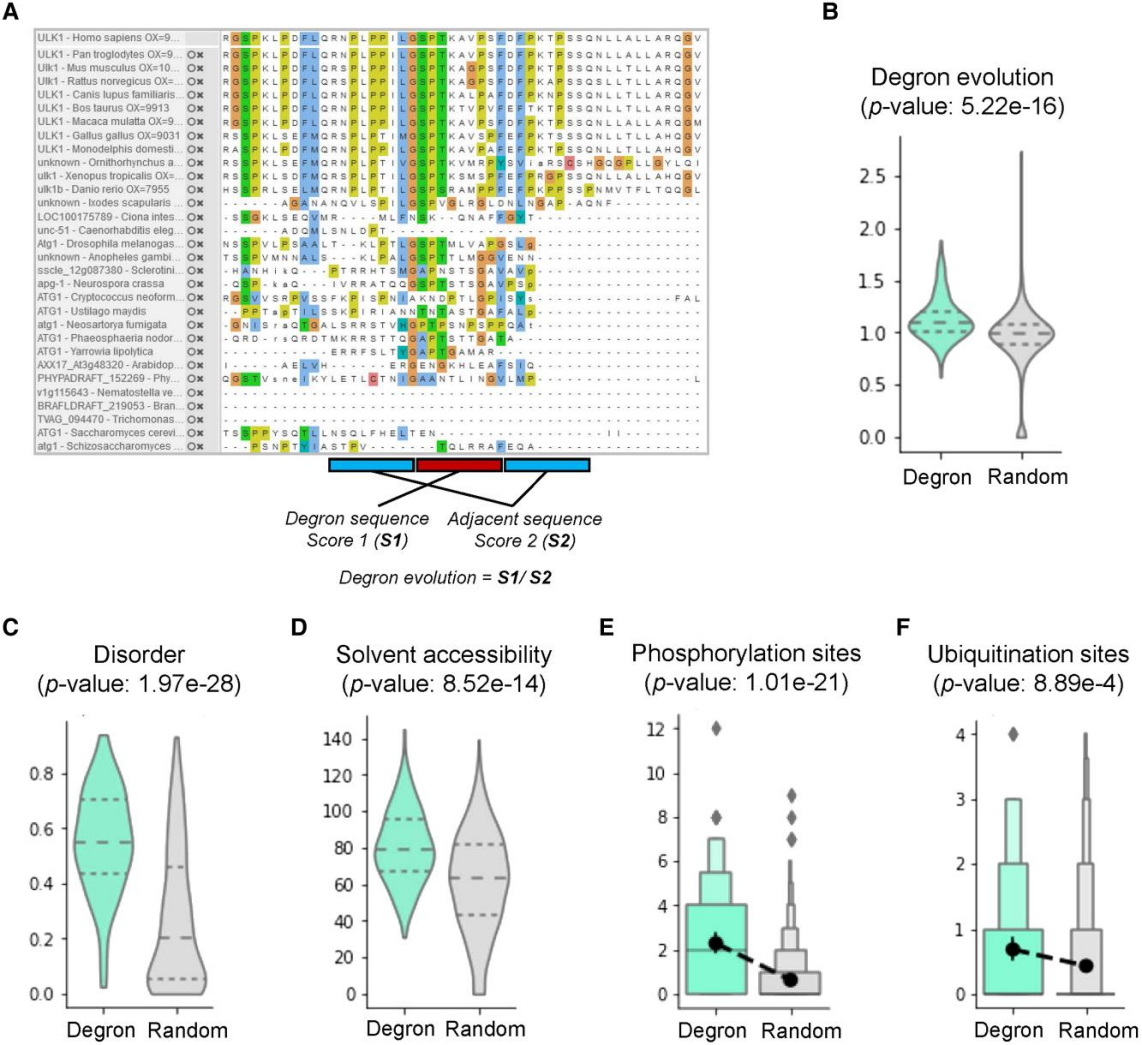
Evolutionary and structural analysis of annotated degron instances and equally long randomly chosen sequences from the human proteome. The *P*-values were derived from 2-tailed Mann–Whitney tests. a) The diagram of degron evolutionary analysis. b) Distribution of the evolutionary scores of annotated degron instances randomly chosen sequences. c, d) Distribution of the disordered c) and solvent-accessible d) scores of annotated degron instances randomly chosen sequences. e, f) The number of phosphorylation e) and ubiquitin conjugation sites (ubiquitination, f) for annotated degron instances and randomly chosen sequences.

**Fig. 3.**
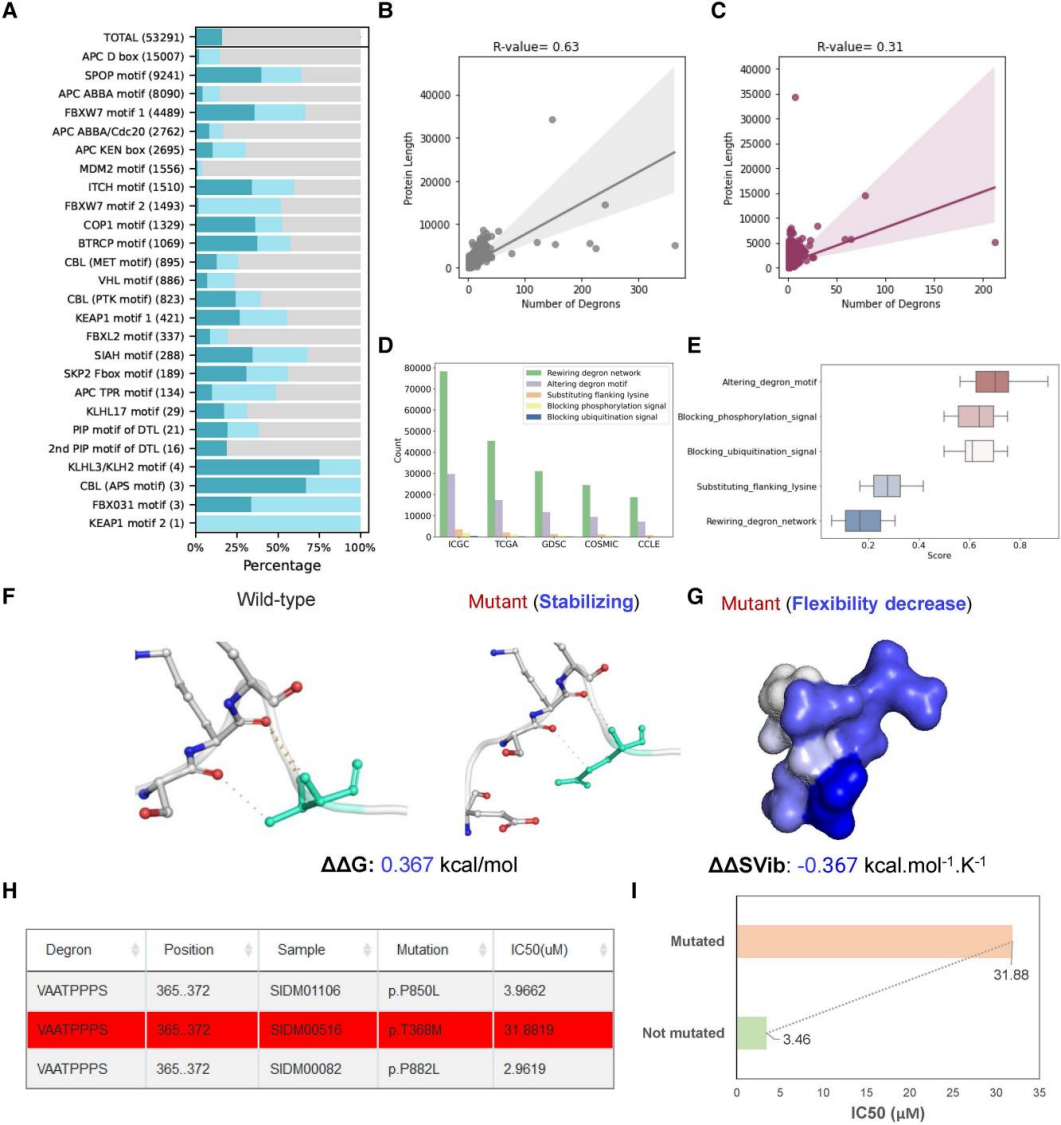
Analysis, validation, and summary for degrons, mutations, and drug responses. a) Statistics of the identified degron instances for each degron motif. b, c) The correlation between the number of degrons and protein length for motif matches b) and multimodal machine learning prediction c). d) The number of degron-associated mutations from 5 cancer-related databases or data sets. e) Distribution of scores for 5 functional outcomes of degron-related mutations. f, g) Analysis of protein stability and flexibility by mutations on degrons. h, i) Potential drug resistance events caused by mutations in functional degrons.

Based on the curated data set, we extracted degrons and their adjacent sequences, which were unified into 20-amino-acid-length fragments. The motif analysis was performed by calculating amino acid preferences at different locations. Sixteen representative motifs were generated characterized by a specific sequence pattern, indicating the specificity of E3 ligase to recognize substrate for degradation (supplementary fig. S1, Supplementary Material online). We assessed the conservation of degrons relative to their neighboring amino acids (Fig. 2a) and found that degrons were evolutionarily conserved (Fig. 2b, *P* = 5.22E−16). We evaluated the enrichment of important PTMs around degrons and examined their structural properties. The results indicated that degrons preferentially occurred in disordered (Fig. 2c, *P* = 1.97E−28) and higher solvent-accessible protein areas (Fig. 2d, *P* = 8.52E−14). Additionally, phosphorylation (Fig. 2e, *P* = 1.01E−27) and ubiquitination signals (Fig. 2f, *P* = 8.89E−4) were significantly enriched in and around degrons. We downloaded a total of 20,377 human proteins that were carefully curated in UniProt database (2021). Sequencing matching of the 23 collected degron motifs within the human proteome yielded a total of 53,291 potential degrons. However, many of these were potentially false positives (Fig. 3a). To address this issue, for each match, we calculated its multiple evolutionary, structural, and physicochemical properties, including disorder, solvent accessibility, secondary structure (coil, helix, and sheet), rigidity, stabilization, flanking conservation, structural domains, number of phosphorylation and ubiquitination sites, and flanking lysine distribution. All matches measured by multimodal features were evaluated by DegML trained on known degron instances and ranked according to the degron probability (Fig. 1a, supplementary fig. S2, Supplementary Material online). Through this approach, we obtained 18,007 degrons (with a threshold greater than 0.5; supplementary table S2, Supplementary Material online). Among them, 8,482 degrons exhibited probabilities exceeding the lowest score observed among verified degrons in their respective classes. These degrons are suggested with high priority for further experimental validation (Fig. 3a). The correlation between the match number and protein length decreased from 0.63 to 0.31 (Fig. 3b and c), indicating that DegML can effectively reduce the false-positive rates.

### Mutational Landscape on Degrome in Pan-cancer

By integrating 5 large cancer mutation data sets and subsequently mapping them to all degrons, we obtained 288,345 somatic nonsynonymous mutation records on 122,503 unique amino acid positions (ICGC: 114,006, TCGA: 66,228, GDSC: 44,874, COSMIC: 35,937, and CCLE: 27,300; Jia et al. 2021). Mutations that intersect with degron regions exhibited varying effects on the degrons. We annotated these mutations by classifying them into 5 types based on their functional effects on degrons (supplementary fig. S3a, Supplementary Material online): rewiring degron network (197,981), altering degron motif (75,453), blocking phosphorylation regulatory signal (8,739), substituting flanking lysine (5,126), and blocking ubiquitination regulatory signal (1,046; Figs. 1b and 3d). Additionally, we developed a method named DegMF to quantify the impact of mutations on the functionality of a degron using a similar strategy previously reported (Martínez-Jiménez et al. 2020), which relies on the observation of phenotypic changes induced by missense mutations (supplementary fig. S3b, Supplementary Material online). Mutations were initially assigned varying weights based on their impact on degrons, with additional consideration given to their impact on essential biochemical properties. Subsequently, all scores were summed and then normalized to the range of 0 to 1. A mutation with a high normalized score exerts a more significant influence on the degradation of the protein in which it is located (Fig. 1c). Following the assessment of the functional impact of each mutation on specific degron using DegMF (supplementary table S3, Supplementary Material online), we uncovered 89,318 actionable mutations surpassing the threshold of 0.5. These mutations are expected to directly influence UPS signaling. The remaining alterations have the potential to perturb the E3–degron network. The result also indicated that mutations mainly alter the essential residues within the degron motifs or eliminate adjacent PTM regulatory sites, leading to dysregulation of degradation signals (Fig. 3e). We randomly selected and validated our predictions by analyzing the effects of actionable mutations on protein stability and physicochemical properties with DynaMut (Rodrigues et al. 2018). As an example, we discovered that the V29E mutation, with a score of 0.66, in the kinase BUB1B could enhance protein stability (Fig. 3f) and decrease protein flexibility (Fig. 3g) via disrupting its degron.

### Landscape of Drug Resistance Mutations in Degrome

To systematically study the degron-related mutations associated with drug response in cancers, we processed raw drug response data from the CCLE and GDSC databases along with cell line annotation files. We exclusively considered drug treatments that target on degron substrates for in-depth analysis. We obtained a total of 326,833 drug treatment records, with each record representing 1 instance in any cancer type. To explore drug resistance due to degron mutations, we initially classified all CCLE and GDSC cell lines according to cancer type. Subsequently, we separated the cell lines into 2 groups for each drug by each cancer type: Group 1 (G1) consisted of cell lines with degron mutations, while Group 2 (G2) included the cell lines without degron mutations. We defined a treatment as a drug–degron–cancer type combination and performed the analysis for each treatment individually. Drug resistance was characterized by the average IC_50_ of G1 surpassing that of G2, as illustrated in Fig. 1d. This process resulted in 432 treatments as potential drug resistance events by comparing the average IC_50_ values of each treatment on the 2 sets of cell lines (supplementary table S4, Supplementary Material online). As an illustration, in SKCM cell lines treated with poly(ADP-ribose) polymerase (PARP1) inhibitor (PARP_9495), the cell lines without any mutation in degron (VAATPPPS) of PARP1 had an average IC_50_ value of 3.46 μM, whereas the cell lines with T368M mutation had an IC_50_ of 31.88 μM. T368M was identified as a drug resistance mutation (score: 0.853) altering degron motif (VAAMPPPS) in PARP1 (Fig. 3h and i).

### The Utility of DegronMD

#### Browse Options

The browsing function allows users to access all the curated and processed data. The available options include all the degrons and their corresponding substrates, the mutational landscape within degrome, the drug response data associated with the substrates and degrons, the network-rewiring events in UPS pathway caused by mutations, and the potential drug resistance outcomes in degron mutation hotspots. On the home page, 5 quick-access buttons are available for conveniently accessing these data sets (supplementary fig. S4, Supplementary Material online). Notably, we provide degron classifications and the introductory details for each degron, available in both a tree view and a data table format. Within the tree view of degron classifications, users can select the specific item to display the detailed annotation information for the corresponding category. In each browsing interface, users can search for the matching records by entering keywords in the top right of the table. Links to each degron and its associated substrates are provided in the last column.

#### Search Options

DegronMD supports 3 search modes: general search, batch search, and BLAST search. Users can conveniently enter 1 or more keywords to query the database. In a general query, users can directly search the DegronMD database by selecting a keyword type and inputting a keyword. For example, if the keyword “ULK1” under type “Gene Symbol” is submitted, the matching results are shown in a tabular format, including gene symbol, protein name, UniProt ID, degron hit, degron classification, degron start and end positions, and the E3 ligase. Detailed annotations of degrons can be accessed through the “Details” link (supplementary fig. S5, Supplementary Material online). In batch search, users can enter multiple keywords, such as gene symbol, protein symbol, UniProt ID, degron hit, and the E3 ligase in a line-by-line format for querying (supplementary fig. S6, Supplementary Material online). The blastp program of NCBI BLAST packages was implemented in the backend of the website. For the BLAST search, users can enter a protein sequence in FASTA format to find similar or homologous proteins. For example, when the protein sequence of the “ULK1” and user-defined “E-value” is submitted, matching homologous proteins exceeding that threshold will be presented in a tabular format (supplementary fig. S7, Supplementary Material online).

#### Annotations for Degrons

The annotation page initiates with the fundamental substrate details, including the gene and protein symbols, multiple cross-referencing IDs, and an interactive 3D view of degron region. Subsequently, 7 tabs are available for presenting extensive annotations on the page.

**Fig. 4.**
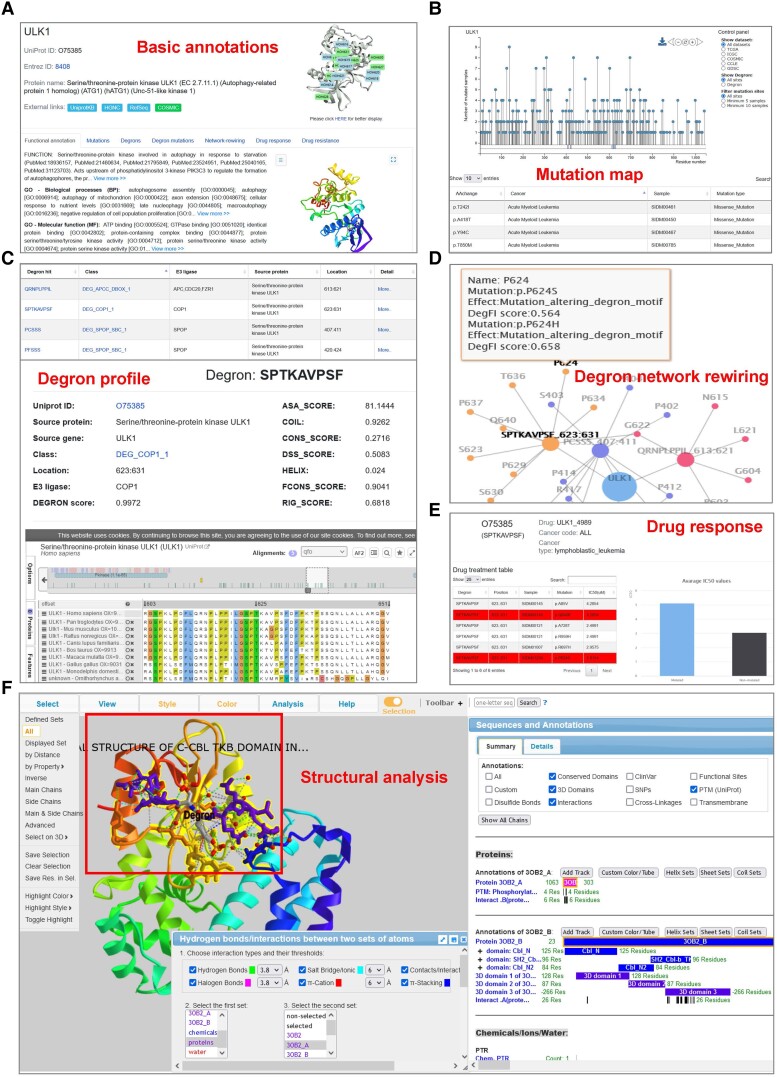
DegronMD database showing annotation pages for degrons, mutations, and drug responses. a) Basic annotation information for the degron substrates. b) Needle plots dynamically show all cancer mutations falling on the selected substrates and their degrons. c) All known and newly identified degrons for the selected substrate are listed with multiple calculated structural and physicochemical properties. d) The network shows E3–degron regulatory network affected by the mutations. e) Drug treatment information and drug resistance event calculated from drug response data. f) 3D protein structure portal for users to explore structural feature of degrons.

(1) Functional annotations: A functional description of a given substrate is included, along with the relevant Gene Ontology (GO) terms. The 3D structural view is provided for users to check and edit the spatial properties of degrons in the substrate (Fig. 4a).

(2) Mutational landscape: This page presents the mutation information associated with the chosen substrate and its degrons. The sample counts at the mutation sites are shown in the needle plot. The map dynamically adjusts as users choose a specific mutation data set through the control panel on the right, allowing options like mutation resource, mutations within degron areas, or filtering based on the number of mutated samples. A comprehensive table listing mutations related to the selected substrate across the 5 cancer-related data sets can be found at the bottom (Fig. 4b).

(3) Degron information: All known and identified degrons for the selected substrate are listed in a tabular format, including degron sequence, degron classification, the E3 ligase, source protein, and degron start and end positions. Detailed annotations of degron can be retrieved through the “More” link. A calculated confidence score and various structural and physicochemical properties for the selected degron are provided. Moreover, an interactive exploration tool is implemented for investigating the functional and evolutionary features of degron (Fig. 4c).

(4) Mutation in degrons: All processed mutations related to degrons of the given substrate are listed in this tab.

(5) Network rewiring: Quantitative analysis of the effect of each mutation on degron is displayed on this page. Users are provided with an intuitive view of the primary hotspots for degron mutation through a global regulated dynamic network. In a bar plot, several diverse impacts of mutations on degron are enumerated. A detailed table is provided to check the consequence from a specific mutation (Fig. 4d).

(6) Drug response: All drug treatment data associated with the given substrate are listed in different cancer types in multiple cell lines.

(7) Drug resistance: In this tab, users can explore potential treatment resistance identified, which is linked to mutations in degrons. Information about drugs and cancer types related to resistance is available. Additional details about drug resistance can be accessed by clicking the “more” tab (Fig. 4e). In addition, we have implemented the port for 1-click structural analysis; with this function, users can easily analyze and visualize degron structures (Wang et al. 2020; Fig. 4f). All the plots (e.g. needle plots, bar plots, and network plots) generated in the database can be downloaded by clicking the “Download” button with multiple formats. We provide the links for users to fully access our processed data on the “Download” page on the website.

## Discussion

There has been a growing interest in the study of targeted protein degradation. Understanding the mechanisms underlying protein degradation is essential for studying cellular processes and developing therapeutics for various diseases (Schapira et al. 2019). However, for most cellular proteins, the degradation mechanisms are poorly understood. Since the UPS system predominantly mediates over 80% of intracellular protein degradation in cells, identifying the key elements that integrate the UPS is essential. In recent years, the field of protein degron research has received considerable attention, focusing on identifying specific degradation signals within proteins and their interactions with E3 ubiquitin ligases (Bondeson et al. 2022; Linster et al. 2022; Miklánková et al. 2022; Heo et al. 2023). To have a better understanding of protein degradation selectivity, researchers have developed high-throughput experimental technologies (Zhang et al. 2023) that combine proteomics (e.g. GPS-peptidome) and computational methods, including machine learning algorithms and protein sequence analysis (Hou et al. 2022).

Despite the growing studies on degrons and their functions, there are only a few bioinformatic resources available for degron screening and analysis. In 2013, the APC/C degron repository was created to provide data on the sequence determinants of the 3 major classes of APC/C degrons (He et al. 2013). In 2017, an interactive web table was released, which listed degron motifs based on data from the ELM resource, supplemented by manual curation (Mészáros et al. 2017). In 2022, Hou et al. developed an online resource that uses a deep learning model to predict proteome-wide degrons and their interactions with E3 ubiquitin ligases (Hou et al. 2022). Additionally, Szulc et al. (2022) introduced DEGRONOPEDIA, a user-friendly web server that allows for screening known degron motifs in protein sequences or structures. This resource also includes the information of PTMs and known pathogenic mutations within the degron sites and their neighborhood sequences. These studies have made valuable contributions to the field of protein degron research. However, existing resources have limitations. Some resources provide only a small number of degron instances and annotation information, while others rely solely on direct motif matching or sequence-based machine learning models to predict results. Furthermore, these resources do not integrate and analyze multiple omics data based on their annotated or identified degrons.

In this study, we aimed to develop the DegronMD to fill this gap. DegronMD represents an integrative knowledgebase available for degron instances across the human proteome for degradation, the mutational landscape on degrome, and related drug response, including drug resistance. We comprehensively identified and annotated over 18,000 new degrons in the human proteome by incorporating consensus motifs with multiple degron-derived features. Since a degron is transferable, i.e. transplanting degron into targeted protein accelerates its degradation, the global map of degron instances for E3 ligases discovered in this work will enable the creation of novel PROTACs and molecular glues to tackle traditionally undruggable proteins (Guenette and Potts 2023).

In addition, degron dysfunction caused by mutations disrupts the protein homeostasis. It is closely associated with diseases such as human cancer, and it can affect the result of therapeutic treatment or possibly inducing drug resistance (Krönke et al. 2014; Liu et al. 2021). By quantifying the impact of mutations on the functionality of degrons, we assembled a mutational atlas on degrome in pan-cancer, which included 89,318 dysfunctional degron events caused by actionable mutations. Mutations in degrons may result in abnormally high gene and protein expression, potentially leading to disease development. Through an integrative analysis of gene expression and DNA methylation profiles from the TCGA, we identified 35 potential driver genes with degron dysregulation and with significantly upregulated expression or downregulated methylation level in different cancer types. Survival analysis was performed to verify the clinical significance and potential therapeutic value of these identified genes. As an illustration, multiomics analysis unveiled *MUC16* and *SLC24A2* as potential driver genes in bladder cancer, while high expression levels of these genes were significantly linked to a shorter survival period, suggesting their potential as therapeutic targets (supplementary fig. S8, Supplementary Material online). Our degron-based analysis offers a novel perspective for elucidating the underlying mechanisms of cancer mutations in tumorigenesis and for identifying potential cancer drivers. Ultimately, through affecting degron, mutations may decrease drug sensitivity or lead to drug resistance by influencing the expression and structural conformation of drug targets. We 7also identified a spectrum of drug resistance–associated mutations in functional degrons; such information may be useful for drug development. To the best of our knowledge, DegronMD is the first knowledgebase for protein-targeted degradation, mutation, and drug response, allowing researchers to investigate the biological mechanisms and implications of protein degradation and screen potential therapeutic targets.

There are some limitations in this study. Firstly, our method for identifying degron instances relies on the presently available data sets, which remain limited. We have identified a number of new degrons through pattern matching and computational predictions. Nevertheless, our method cannot predict protein terminal degrons and other degrons of those E3 ligases that were not in the training data set. In addition, within a protein, it is reasonable to hypothesize that the relationships between different degrons exhibit variability, with some functioning independently (different E3 ligase binding) and others acting cooperatively (same E3 ligase binding) to enhance degradation efficiency. A notable example of degron cross-talk is exemplified by the interaction between the substrate NRF2 and KEAP1. NRF2 contains 2 degrons located in close proximity, and their concerted action mediates binding to a KEAP1 dimer, which is pivotal for the stabilization and activation of NRF2 in response to electrophilic stress (McMahon et al. 2006). However, the direct targets of most of the nearly 600 E3 ligases are unknown. Even for well-characterized E3 ligases, additional unknown partners may exist. So, we are currently unable to explore the cross-talk before degrons. More experimentally validated degrons and corresponding E3 ligases are needed to make a more accurate and comprehensive model to identify degrons and their cross-talks. Moreover, when additional 3D structures of degron with cognate E3 ligases are released in the future, more structure-based characteristics should be considered to distinguish functional degrons. Finally, experimental validation will be important for verifying specific degrons of interest and their cognate E3 ligases predicted by the model. Such results will not only enhance our model and prediction performance but also lead to novel discovery. However, as a computational research lab, we are not able to perform such validation in the near future, which is also labor intensive and expensive. Instead, we will frequently search the most recent literature to find such data to support our prediction. Accordingly, DegronMD will be subject to continuous maintenance and be updated with the new data and results in the future.

## Supplementary Material

msad253_Supplementary_Data

## Data Availability

Researchers can access DegronMD online at https://bioinfo.uth.edu/degronmd. All the data sets generated in this study and through online analysis can be downloaded by clicking the “Download” button at https://bioinfo.uth.edu/degronmd/download.html.
