# Supplementary material for: DegronMD: Leveraging Evolutionary and Structural Features for Deciphering Protein-Targeted Degradation, Mutations, and Drug Response to Degrons
Source: Mol Biol Evol. 2023 Nov 22;40(12):msad253. doi: 10.1093/molbev/msad253 (PMC10701100; doi:10.1093/molbev/msad253)
Supplement: msad253_Supplementary_Data [file msad253_supplementary_data.zip › Supplementary material.docx]

**Supplementary material for “****DegronMD: Leveraging evolutionary and structural features for deciphering protein-targeted degradation, mutation and drug response to** **degrons”**

**Supplementary Figure**


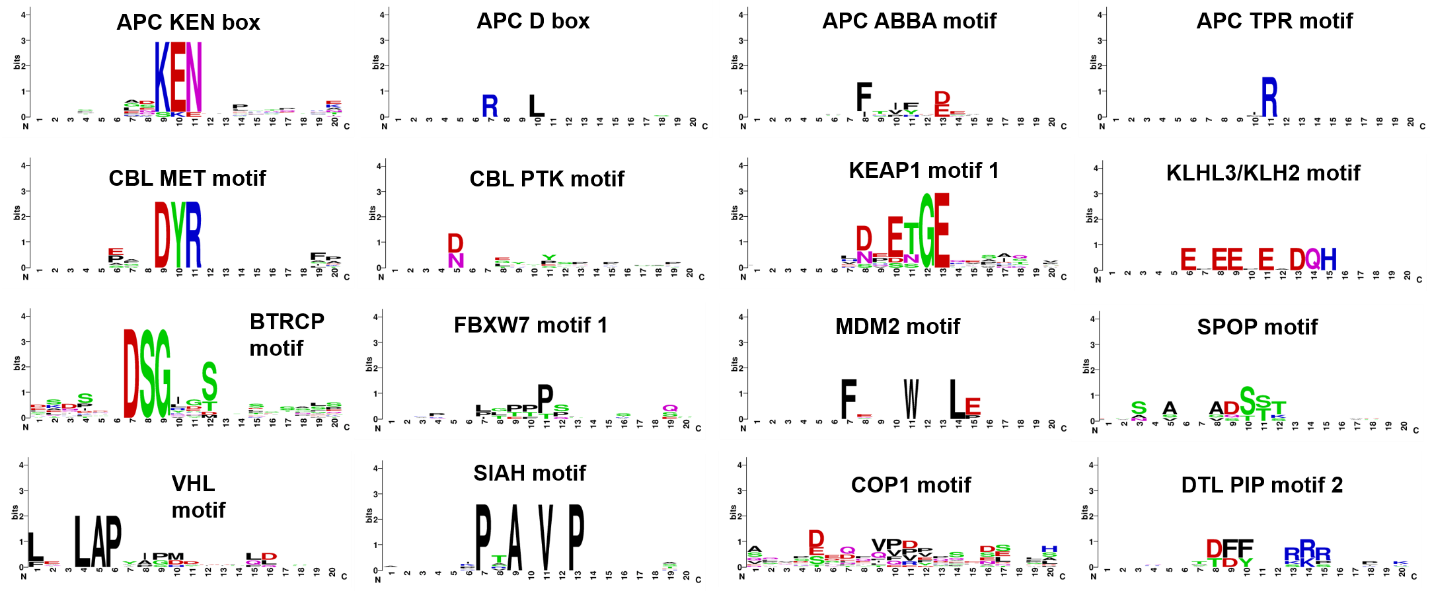


**Supplementary Figure 1**. The amino acid preferences at different locations and the logo plots of different degron instances.

**
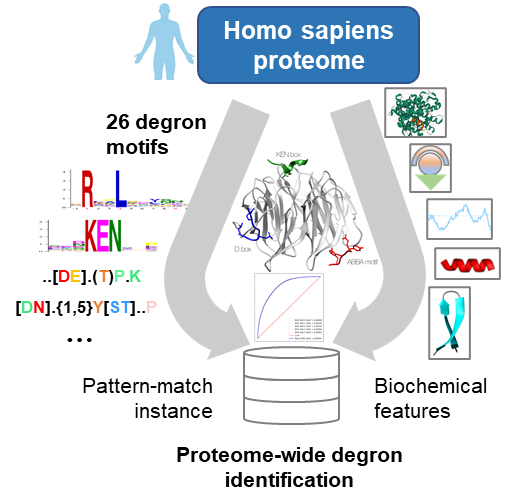
**

**Supplementary Figure 2**. DegML for the identification of degrons in human.

**
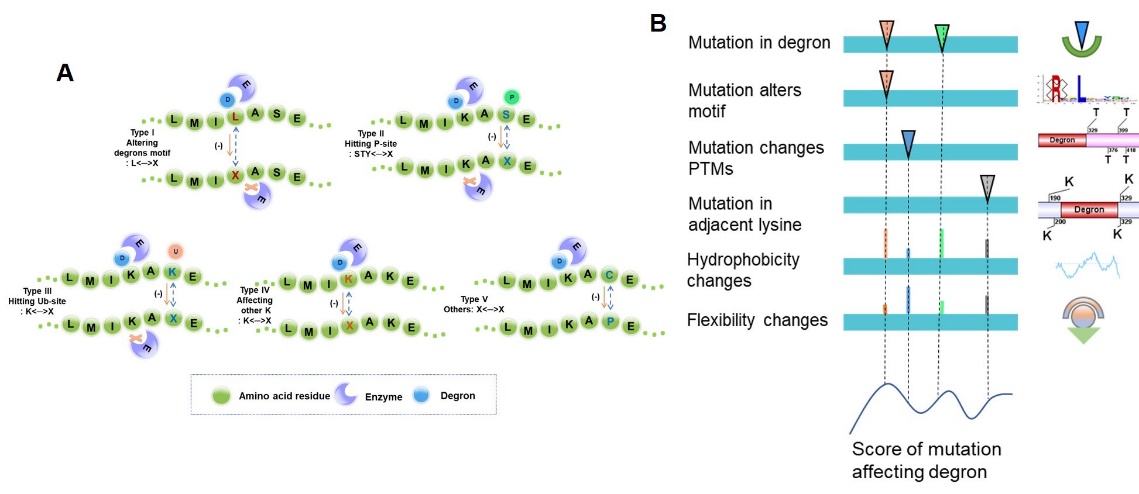
**

**Supplementary Figure 3**. **A**. All mutations were classified into five types according to their functional outcomes for degrons. **B**. DegMF for assessing the impact of mutations on the functionality of a degron.

**
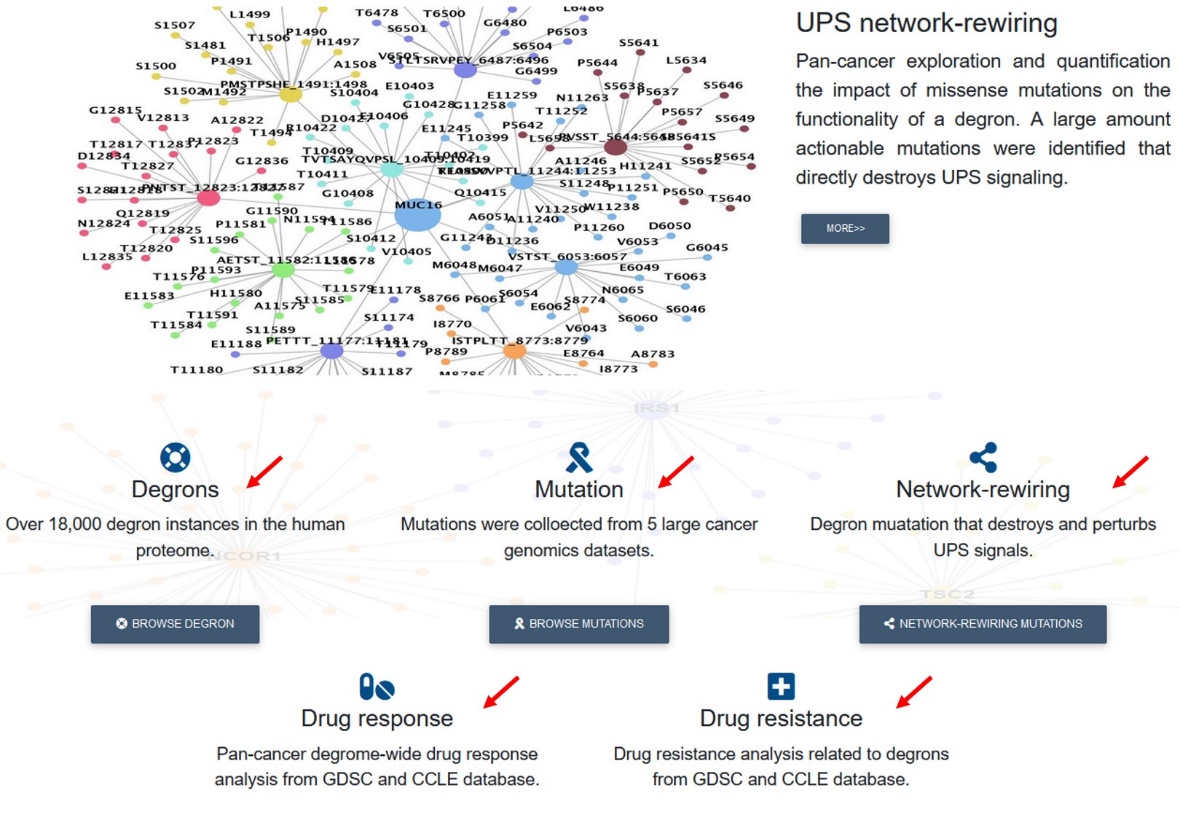
**

**Supplementary Figure 4**. The browse function allows users to view all the curated and processed data.

**
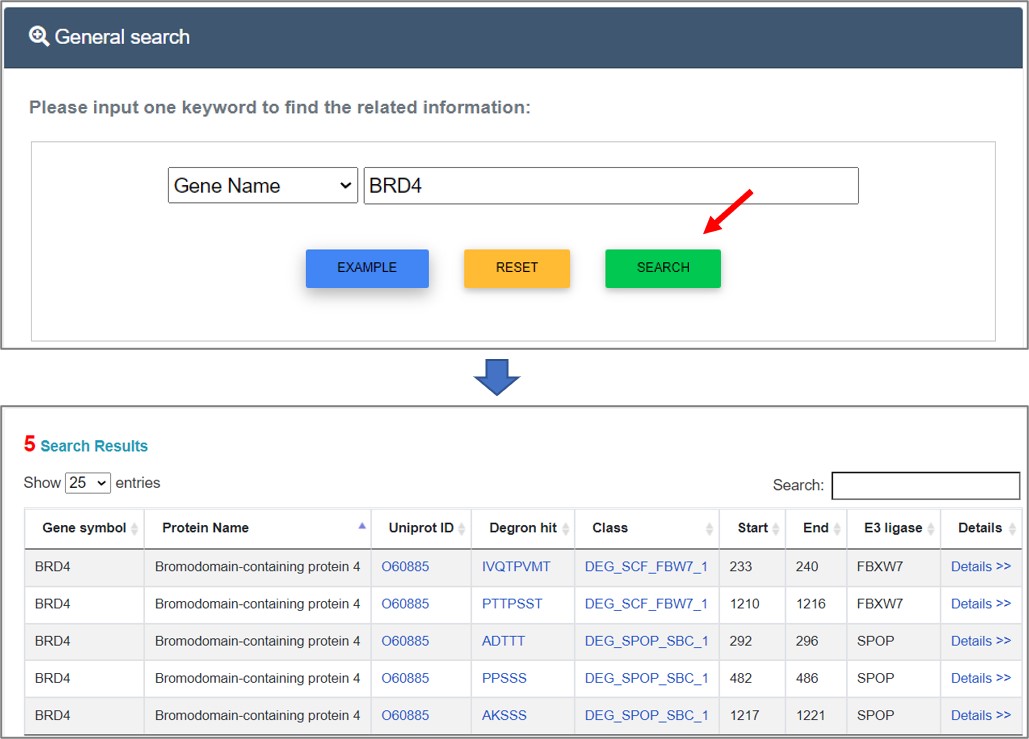
**

**Supplementary Figure 5**. General search option in DegronMD.

**
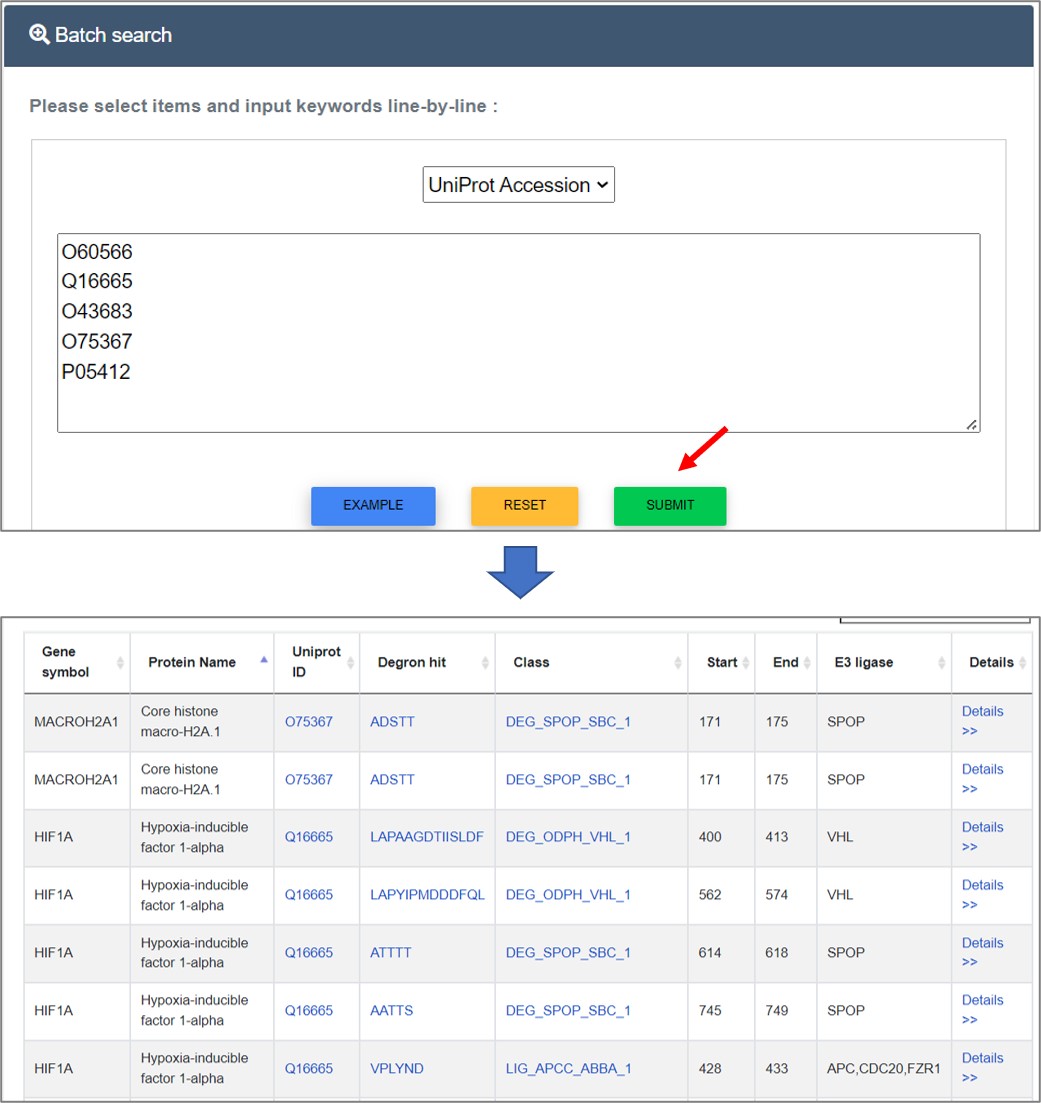
**

**Supplementary Figure 6**. Batch search option in DegronMD.

**
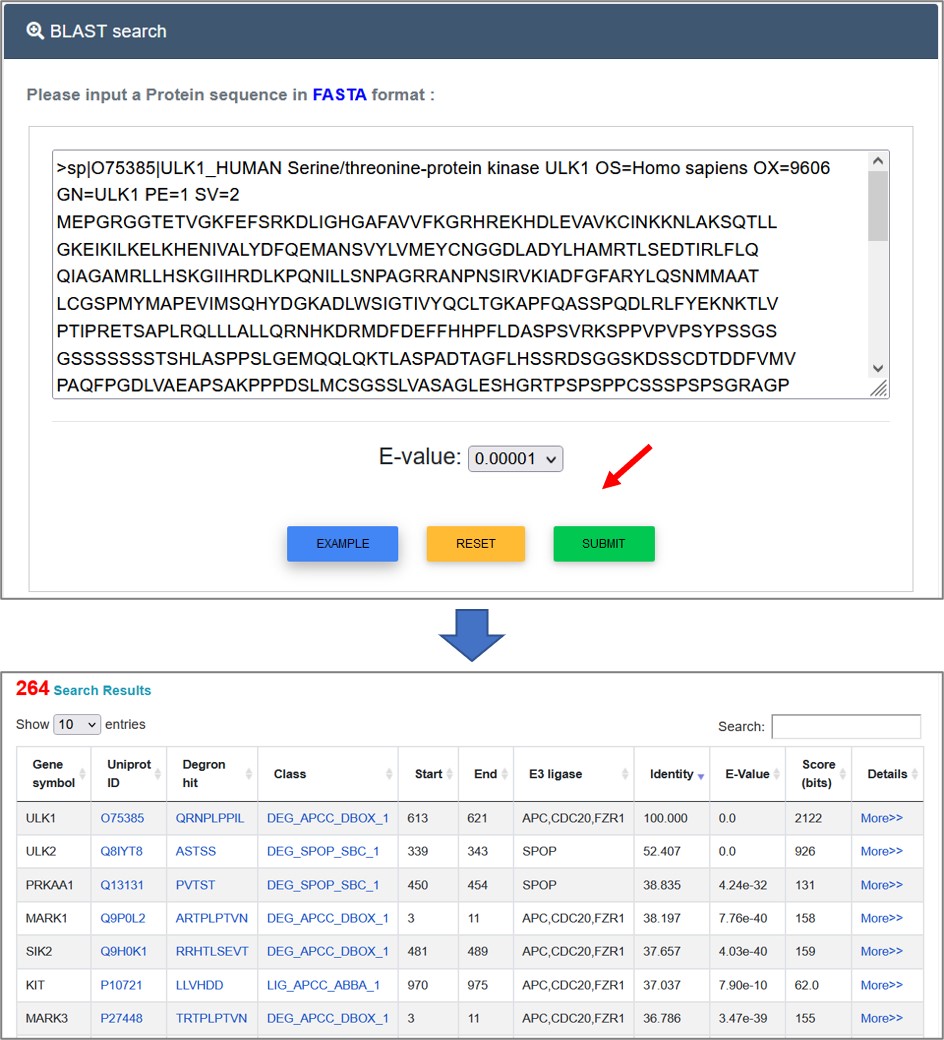
**

**Supplementary Figure 7**. BLAST search option in DegronMD.


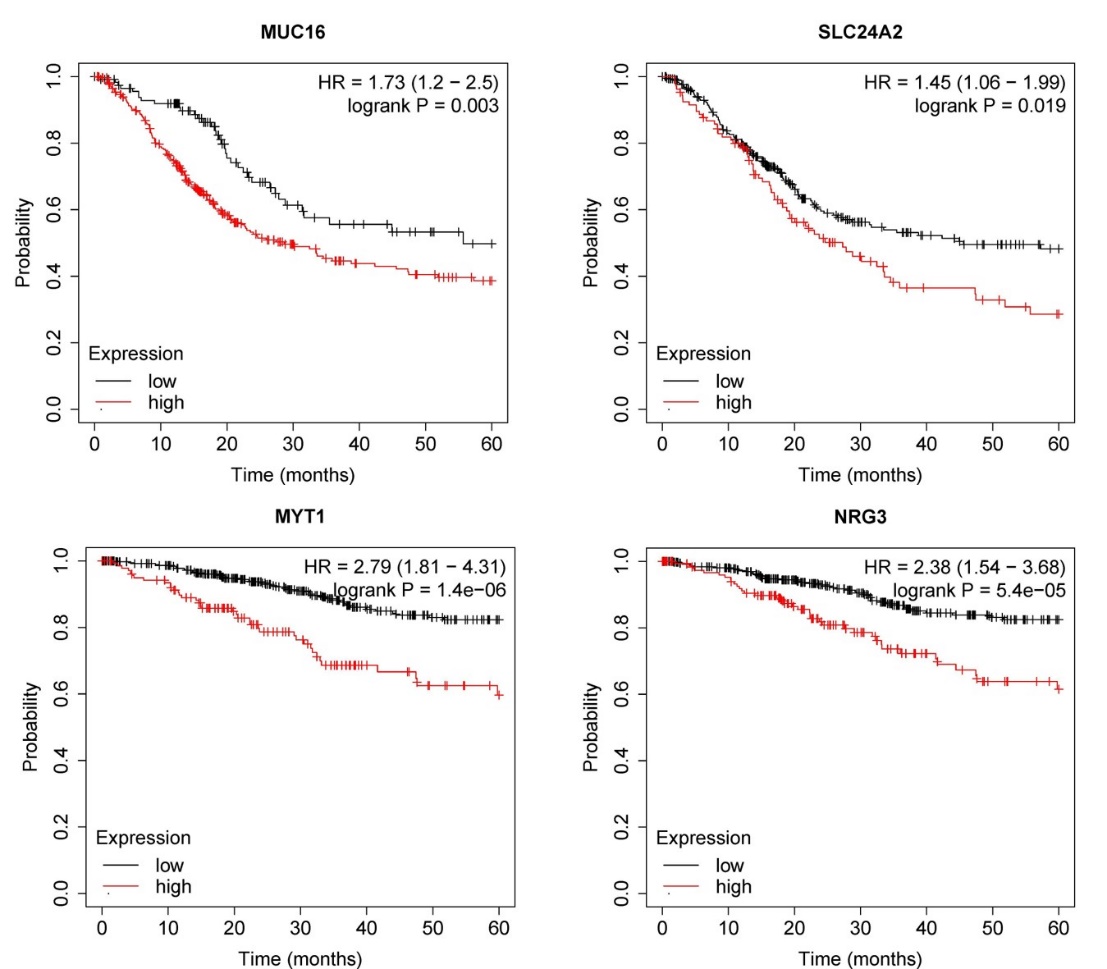


**Supplementary Figure 8**. Kaplan-Meier estimates of overall survival based on expression of the signature gene identified in multi-omics analysis.
